# Prediction of Compressive Strength of Concrete Considering Pore Relative Humidity

**DOI:** 10.3390/ma18122859

**Published:** 2025-06-17

**Authors:** Gyeonghee An

**Affiliations:** Korea Atomic Energy Research Institute, Daejeon 34057, Republic of Korea; akh425@kaeri.re.kr

**Keywords:** concrete, compressive strength, relative humidity, self-desiccation, diffusion, degree of hydration

## Abstract

This study investigates a prediction method for the compressive strength of concrete considering the pore relative humidity. Water within concrete not only facilitates the bonding of cementitious materials and aggregates but also influences the pore structure, thus affecting the compressive strength of concrete. While the relationship between the water–cement ratio and mechanical properties has been extensively explored, the quantitative effects of curing and moisture history on compressive strength remain insufficiently demonstrated. This research aims to fill this gap by proposing predictive models that consider the history of pore humidity. Experimental data from previous studies were utilized to develop and verify these models. Pore humidity was assessed through self-desiccation and diffusion processes. A self-desiccation model was formulated based on existing experimental results, and the finite element method was employed for diffusion analysis. The prediction model for compressive strength was derived from the rate constant model, incorporating apparent activation energy and adjusting for various curing conditions. The proposed models provide a robust framework for predicting the compressive strength of concrete under diverse curing scenarios. This research contributes to the development of practical tools for ensuring the safety and durability of concrete structures in the construction industry.

## 1. Introduction

Water in concrete is essential to bond the cementitious materials and aggregates together and form compounds such as C-S-H gel, which makes the concrete hard. Water also affects the pore structure of concrete. Therefore, moisture inside the concrete is strongly related to the development of its mechanical properties, such as compressive strength.

Many researchers have studied the effect of moisture on the compressive strength of concrete. Various experiments have been conducted to demonstrate the relationship between the water–cement ratio and compressive strength, and corresponding models for prediction have been suggested. It is known that the compressive strength is higher when the water–cement ratio is lower.

Both the initial amount of water for mixing and the curing method to maintain moisture are important factors. Although the term ‘curing’ typically refers to processes involving various temperatures, in this paper, it specifically denotes moisture-related curing methods—such as moist, sealed, or dry curing—conducted at a standard temperature. Compressive strength is highly dependent on the curing method and duration, especially at an early age. Therefore, standards in many countries require a minimum period of moist curing to ensure strength for safety. Various curing methods have been developed for practical use in the construction field. However, some construction sites may have very dry conditions, and others may not have a long enough curing period due to their schedule. Additionally, moisture content in a massive structure can vary depending on the position, and the curing condition can also vary inside the structures.

Despite all of the research, the effects of curing and of the corresponding history of moisture in concrete on compressive strength have not been quantitatively demonstrated, unlike the effect of temperature. Several models predict strength depending on temperature, but no general model exists for humidity. The reasons are the difficulty in measuring humidity and the various factors affecting it. Measuring the change in pore humidity inside concrete is more complex than measuring temperature. Sensors for detecting humidity are less accurate and stable compared to thermocouples, especially under very humid conditions. Also, humidity should be measured at several points because there exists a gradient inside the concrete, and controlling ambient humidity in the laboratory is much harder.

The importance of considering humidity for predicting compressive strength is increasing because the types of structures and environmental conditions are diversifying. Modern structures are becoming much bigger and taller and are exposed to various climates due to the globalization of the construction business. Special structures, such as nuclear power plants, need to be controlled more accurately for safety. Therefore, the correlation between the compressive strength of concrete and pore humidity should be demonstrated, and a prediction model is necessary.

The objective of this study is to propose an appropriate model to predict the compressive strength of concrete considering the history of pore relative humidity. The history of pore humidity inside concrete over time is first defined for certain specimens or structures, and then the compressive strength is analyzed based on this history. Experimental data from previous research are used to suggest and verify the prediction model. Pore humidity is evaluated via self-desiccation and diffusion. A model for self-desiccation is suggested based on experimental results in the literature, and the finite element method is used for diffusion analysis.

The prediction model for compressive strength is based on the rate constant model using the apparent activation energy. The rate constant model is applicable for the standard curing condition, which maintains the pore humidity at 100%. Pore humidity under other curing conditions differs from that of standard curing. Compressive strength can be analyzed by adapting the developed relationship between pore humidity and the rate of hydration. The rate of hydration decreases as humidity drops, affecting the compressive strength of concrete.

## 2. Prediction of Pore Humidity

Moisture in concrete is one of the main factors that affects not only the mechanical properties but also many other properties, such as long-term deformation, thermal conductivity, and crack formation. Some portion of the moisture is used for a chemical reaction called hydration, and the rest evaporates or diffuses over time. It is obvious that determining the history of the moisture, often expressed in terms of relative humidity in the pores, helps predict concrete properties.

The decrease in moisture due to hydration is called self-desiccation and is affected by several factors, such as the water–cement ratio, the type of cement, and the addition of silica fume. It is known that self-desiccation is of great importance to high-performance concrete, especially with a water–cement ratio below 0.39 [1], and the internal humidity drop due to self-desiccation is normally not over 25% [2,3]. Summaries of experimental results and suggested models for self-desiccation are described in Section 2.1.

Diffusion also affects pore humidity in concrete. Diffusion in concrete is very complex due to the wide range of pore sizes and the change in pore structure with age [4]. Governing equations for the drying and wetting of concrete, formulated by Bazant [5] are widely used for diffusion analysis. According to the equations, diffusion in concrete can be characterized by the diffusion coefficient, surface factor, and ambient condition. Research [5,6,7] shows that the diffusion coefficient is a function of pore humidity, making diffusion a nonlinear problem. Governing equations for diffusion and the formulation for the finite element method are briefly introduced in Section 2.2.

### 2.1. Self-Desiccation

Most experimental work on self-desiccation is performed by measuring the internal relative humidity of sealed specimens to prevent moisture diffusion. Several models to predict the internal humidity drop due to self-desiccation have been suggested based on experimental results. The water–cement ratio is the main factor governing the self-desiccation in prediction models.

Paillere et al. [3] measured the relative humidity of various cement pastes and mortar specimens with and without silica fume during sealed curing. The water–cement ratio varied from 0.19 to 0.4, and the portion of silica fume varied from 0 to 25%. The experimental results show that the humidity did not lower below 75%. Persson [1,8,9,10,11,12] conducted numerous experiments and suggested models for the self-desiccation of various concretes. The results also show that self-desiccation depends on the age of the concrete and the water–cement ratio (effective water–cement ratio if silica fume is used). He suggested four types of models [1,10,11] to predict the changes in relative humidity due to self-desiccation based on his experiments. Prediction models are not consistent due to experimental difficulties such as the sealing method or measurement of humidity. Additionally, there are some restrictions on the water–cement ratio or age of concrete depending on the models. Kim and Lee [13] measured the internal relative humidity in both sealed and drying specimens. Three water-to-cement ratios, 0.28, 0.4, and 0.68, were used, and probes from the company Vaisala were used. The experimental results show higher self-desiccation with a lower water–cement ratio. Kim and Lee [13,14] also suggested a simple model for each type of concrete tested. Jiang et al. [15] showed the effects of the water–cementitious ratio, silica fume (SF), and ground blast-furnace slag (GBFS) on self-desiccation and autogenous shrinkage. Four kinds of mixtures were tested by probes. Jiang et al. [15] suggested a model similar to Persson [10] for each mixture experimented. Zhang and Ye [16] conducted experiments on the development of humidity due to self-desiccation in blended cement pastes from 1 day to 1.5 years using Rotronic HygroClip2 sensors (Rotronic, Bassersdorf, Switzerland), which use crushed paste samples. Zhang and Ye [16] suggested another model for each mixture assessed. Zhang and Zhao [17] measured three mixtures of concrete with sensors from Sensirion. Other researchers, such as Shen et al. [18], Wyrzykowski and Lura [19], Chen et al. [20], Zhang et al. [21], Kang et al. [22], and others, measured the relative humidity due to the self-desiccation of various mixtures. The prediction of self-desiccation is directly related to the mechanical properties of concrete under sealed conditions. It is quite important to predict self-desiccation, but there exist some limitations in the existing models summarized.

It is necessary to suggest a better model for self-desiccation, especially for cement of early ages. Therefore, a new model for self-desiccation is suggested through a regression analysis of the experimental results.

The new equation suggested, shown in Equation (1), is similar to the equation for an adiabatic temperature rise curve to model the ultimate value and speed of self-desiccation.

(1) $$h_{s}=1-h_{so}(1-e^{-\alpha_{s}t})$$

where $h_{s}$ is the pore humidity at time t due to self-desiccation, $h_{so}$ is the ultimate pore humidity due to self-desiccation, $\alpha_{s}$ is the speed of the humidity drop per day, and t is the age of the concrete in days.

**Figure 1:**
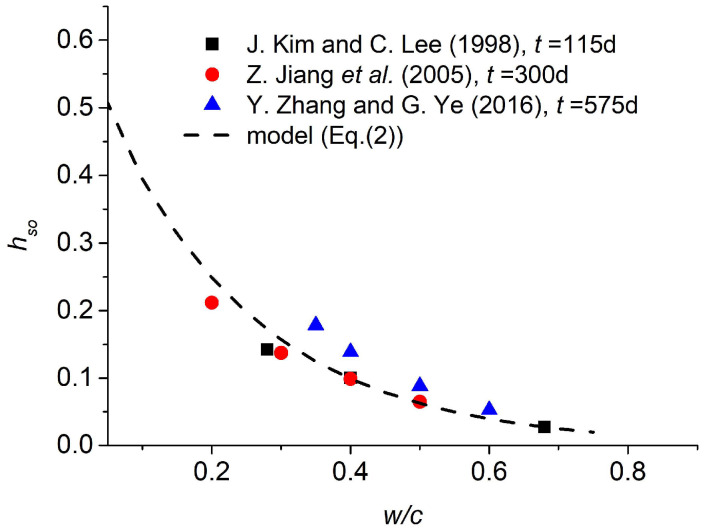
The ultimate pore humidity caused by self-desiccation [14,15,16].

When the water–cement ratio (w/c) increases, the ultimate pore humidity $h_{so}$ and the speed of humidity drop $\alpha_{s}$ decrease. Figure 1 shows the experimental results of the ultimate pore humidity $h_{so}$ from previous research [14,15,16]. The data in Figure 1 were obtained at ages of 115, 300, and 575 days—sufficient to assume ultimate pore humidity.

The relationship between the ultimate pore humidity $h_{so}$ and water–cement ratio can be linear(−A(w/c)+B), power($A{\left(w/c\right)}^{-B}$), or exponential($Ae^{-B(w/c)}$), but the exponential function is the best for fitting, as shown in Figure 1. The power function is the best for speed $\alpha_{s}$. The functions for $h_{so}$ and $\alpha_{s}$ are decided as shown in Equations (2) and (3).

(2) $$h_{so}=a_{1}e^{-b_{1}(w/c)}$$

(3) $$\alpha_{s}=a_{2}{\left(w/c\right)}^{-b_{2}}$$

where $a_{1}$, $b_{1}$, $a_{2}$, and $b_{2}$ are the parameters decided by the regression analysis of the experiments.

The parameters for Equation (2) are $a_{1}=0.625$ and $b_{1}=4.607$, and for Equation (3) are $a_{2}=0.014$ and $b_{2}=1.877$, as a result of a regression analysis of all of the experimental results from previous research. The final suggested equation for pore humidity caused by self-desiccation is as shown in Equation (4), which combines Equations (1)–(3).

(4) $$h_{s}=1-0.6e^{-4.6(w/c)}(1-e^{-0.014{\left(w/c\right)}^{-1.9}t})$$

**Figure 2:**
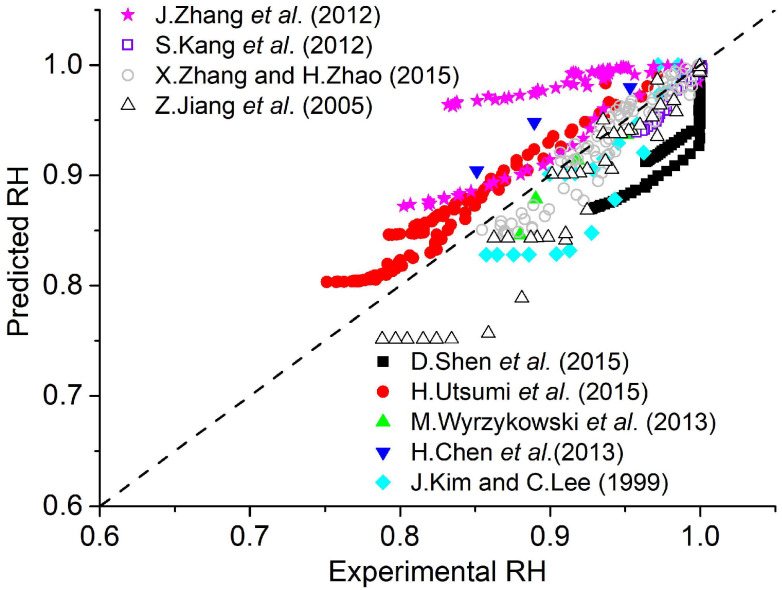
Experiments and prediction of self-desiccation by the new model ($R^{2}=0.655$) [13,15,17,18,19,20,21,22,23].

A comparison of the experimental results and the humidity predicted by the new model in Equation (4) is shown in Figure 2. It shows a better fit, especially for humidity near 1.0, which represents the early ages of concrete, which is very important for predicting the properties of concrete.

**Figure 3:**
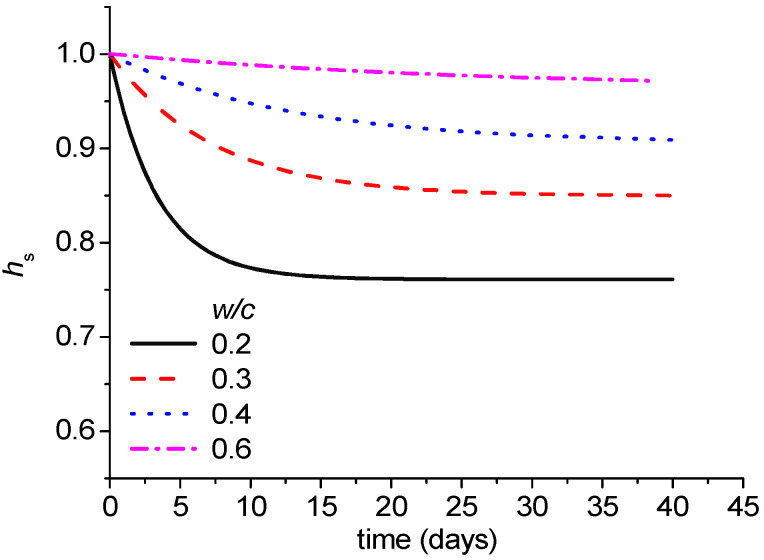
Prediction of pore humidity due to self-desiccation by new model.

Figure 3 shows the prediction of pore humidity by the new model depending on the water–cement ratio. It shows the tendency for pore humidity to drop more and faster when the water–cement ratio is lower.

### 2.2. Diffusion

The governing equation for diffusion and important parameters that affect diffusion, such as the diffusion coefficient and the surface factor, are explained, and the formulation for the finite element method used in this study is briefly introduced herein.

The governing equation and the boundary condition for nonlinear diffusion analysis are shown in Equations (5) and (6), respectively [5,6].

(5) $$\frac{𝜕h}{𝜕t}=div(D\;grad\;h)$$

where h is the pore relative humidity, t is the time, and D is the moisture diffusion coefficient.

(6) $$D{\left(\frac{𝜕h}{𝜕n}\right)}_{sf}=f_{sf}(h_{en}-h_{sf})$$

where n is the unit outward normal at the surface, $f_{sf}$ is the surface factor, $h_{en}$ is the environmental humidity, and $h_{sf}$ is the surface humidity.

The dependence of diffusivity on pore water content was first considered by Pihlajavaara [24] and co-workers. Since then, various models for the diffusion coefficient have been developed. In this research, a diffusion coefficient model for isotherm condition, suggested in the *fib*2010 code [25] and research by Bazant [26,27], as shown in Equation (7), is used for the analysis.

(7) $$D\left(t,h\right)=D_{1}(t)\left(\alpha_{D}+\frac{1-\alpha_{D}}{1+{\left[(1-h)/(1-h_{c})\right]}^{n}}\right)$$

where $D_{1}$ is the maximum diffusion coefficient, $\alpha_{D}$ is the ratio of the minimum diffusion coefficient to the maximum diffusion coefficient, $h_{c}$ is the pore relative humidity at $D=0.5D_{1}$, and n is an exponent. The parameters $\alpha_{D}$, $h_{c}$, and n are suggested in the *fib*2010 code [25] to be 0.05, 0.8, and 15, respectively.

The maximum diffusion coefficient can be defined as follows:

(8) $$D_{1}\left(t\right)=D_{o}f(t)$$

where $D_{o}$ is the diffusivity of hardened concrete in $m^{2}/h$, and f(t) is a function of time.

The diffusivity of hardened concrete can be defined as a function of compressive strength, as suggested in the *fib*2010 code [25]:

(9) $$D_{o}=\frac{D_{1.0}}{f_{ck}/f_{cko}}$$

where $D_{1.0}=3.6\times 10^{-6}m^{2}/h$, $f_{ck}$ is the characteristic compressive strength of concrete (MPa), and $f_{ck}$=10 MPa.

The diffusion coefficient suggested in the *fib*2010 code [25] is for hardened concrete, but the diffusion coefficient at early ages is about 10~100 times higher than that of hardened concrete [26,28]. This indicates that the diffusion coefficient is a function of the age of concrete. In this study, the effect of age on the maximum diffusion coefficient is assumed to be as follows [26]:

(10) $$f\left(t\right)=0.3+\sqrt{13/t}$$

The time function for the maximum moisture diffusion coefficient becomes 1 when t=26.5 days.

The surface factor, which affects diffusion at the surface, is proportional to the water–cement ratio of concrete [6]:

(11) $$f_{sf}=2.17\times 10^{-3}(w/c)-8.56\times 10^{-4}$$

where $f_{sf}$ is the surface factor in m/h, and w/c is water–cement ratio 0.4≤w/c≤0.7.

For the finite element method (FEM), the governing equation, Equation (5), can be rewritten as Equation (12) by the Galerkin method. To solve this equation numerically, the Crank–Nicolson method is used in this study.

(12) $$\left[C\right]\left\{\frac{𝜕h}{𝜕t}\right\}+\left[k\right]\left\{h\right\}=\{q\}$$

where $\left[C\right]$, $\left[k\right]$, and {q} are defined as the following equations.

(13) $$\left[C\right]=\int_{V}^{⁠}{\left[N\right]}^{T}\left[N\right]dV$$

(14) $$\left[k\right]=\int_{V}^{⁠}D\left(\frac{𝜕{\left[N\right]}^{T}}{𝜕x}\frac{𝜕[N]}{𝜕x}+\frac{𝜕{\left[N\right]}^{T}}{𝜕y}\frac{𝜕[N]}{𝜕y}+\frac{𝜕{\left[N\right]}^{T}}{𝜕z}\frac{𝜕[N]}{𝜕z}\right)dV+\int_{S}^{⁠}f_{sf}{\left[N\right]}^{T}[N]dS$$

(15) $$\left\{q\right\}=\int_{S}^{⁠}f_{sf}{\left[N\right]}^{T}h_{en}dS$$

where $\left[N\right]$ denotes the matrix of the shape functions, S is the surface, and V is the volume.

## 3. Prediction of Compressive Strength Considering Pore Humidity

The compressive strength of concrete is affected by the history of pore humidity inside the concrete. However, there are no appropriate models considering pore humidity because it is difficult to conduct controlled experiments related to humidity. While several studies have investigated the effect of the curing relative humidity, most focus on external curing environments. Experimental data are available not only for concrete specimens, but also for mortar and cement paste specimens [29,30]. In addition, studies have been conducted on special types of concrete, such as self-consolidating concrete [31]. Research using artificial intelligence, including a fuzzy-logic-based system [32], has also been actively pursued to predict concrete properties. These studies generally propose humidity-adjusted maturity functions, with parameters calibrated from experimental results [29,30,33]. Most of these models are formulated as a function of curing humidity, not pore humidity. In this section, a new prediction model for compressive strength considering pore humidity is suggested with some appropriate assumptions and analyses.

The prediction of compressive strength considering pore humidity is based on a prediction model called the rate constant model. The compressive strength of concrete cured in water at various temperatures can be predicted by the existing rate constant model, but this model can only consider the effect of temperature on strength under a moist curing condition. It is known that the compressive strength is dependent on the rate of hydration, and the rate of hydration is related to the moisture or humidity condition. Therefore, a model for predicting the rate of hydration depending on pore humidity, and another model for the development of mechanical properties depending on the rate of hydration, are suggested to predict the mechanical properties of concrete cured under various ambient humidity levels. The new model is suggested and verified by experimental results, showing that the suggested model is reasonable.

### 3.1. Rate Constant Model with Apparent Activation Energy

In 1991, Tank and Carino [34] formulated the rate constant model based on a mathematical expression to describe the compressive strength development of concrete. Its derivation follows ideas originally presented in 1956 by Bernhardt [35]. The rate of strength gain at any age is assumed to be a function of the current strength and the temperature:

(16) $$\frac{dS}{dt}=f\left(S\right)k(T)$$

where $f\left(S\right)$ is a function of strength S, and k(T) is the rate constant, which is a function of temperature and has the same meaning as a maturity function.

To improve the rate constant model, Han [36] suggested prediction models based on a new apparent activation energy function. Their derivation follows the same ideas as the rate constant model. The rate of strength gain is the same as in Equation (16). Because the Arrhenius function estimates the effect of temperature more accurately at early ages, Han [36] suggested that the rate constant function k(T,t) should be based on the Arrhenius equation:

(17) $$k\left(T,t\right)=Ae^{-\frac{E}{RT}}$$

where A is a constant of proportionality, E is the activation energy, R is the gas constant, and T is the temperature.

Han modified the apparent activation energy function to be considered as a function of age. If the apparent activation energy is a function of age and Equation (17) is substituted into Equation (16), Equation (16) can be integrated as follows:

(18) $$\int_{0}^{S}\frac{1}{f(S)}dS=\int_{t_{0}}^{t}k\left(T,t\right)dt=\int_{t_{0}}^{t}Ae^{-\frac{E(t)}{RT}}dt$$

Some investigators [37,38] stated that the apparent activation energy changes slightly at early ages and decreases greatly at later ages. The decrease in apparent activation energy can reduce overestimation of the effect of temperature on strength development at later ages [37]. The temperature does not influence strength development after a certain period, and hence, the activation energy eventually approaches zero. Because Jonasson [39] reported that overestimation of the influence of temperature on strength development is greater at high temperatures, the apparent activation energy of concrete cured at a high temperature will decrease more quickly than that cured at a normal temperature. Based on previous findings [37,38,39,40,41], to represent the variation in apparent activation energy, Han [36] proposed the following exponential function:

(19) $$E\left(t\right)=E_{o}e^{-\beta t}$$

where $E_{o}$ is the initial apparent activation energy (J/mole) and β is a constant.

If a hyperbolic equation is used for the strength function [35,40,41] as shown in Equation (20), Equation (18) can be expressed as Equation (21):

(20) $$f\left(S\right)=S_{u}{\left[1-\frac{S}{S_{u}}\right]}^{r}$$

(21) $$\int_{0}^{S}\frac{1}{S_{u}{\left[1-\frac{S}{S_{u}}\right]}^{r}}dS=\int_{t_{0}}^{t}k\left(T,t\right)dt$$

where $S_{u}$ is the strength at infinite time and r is a reaction coefficient.

Moon [40,41] suggested that a reaction coefficient r equal to 3 estimates the later-age strength development more accurately than one equal to 2. Thus, Equation (21) can be integrated and rewritten as Equation (22), expressing the relative strength in terms of 28-day strength $S_{28}$.

(22) $$\frac{S}{S_{28}}=R_{u}\left\{1-\frac{1}{\sqrt{1+2\int_{t_{0}}^{t}k\left(T,t\right)dt}}\right\}$$

where $R_{u}$ is the limiting relative strength with $R_{u}=S_{u}/S_{28}$.

The strength equation can be obtained as follows:

(23) $$\frac{S}{S_{28}}=R_{u}\left\{1-\frac{1}{\sqrt{1+A\left[e^{-\frac{E_{o}}{RT}e^{-\beta t}}+e^{-\frac{E_{o}}{RT}e^{-\beta t_{0}}}\right]}(t-t_{0})}\right\}$$

Equation (23) represents relative strength development under a constant moist curing temperature as functions of five unknown parameters ($R_{u}$, A, $E_{o}$, β, and $t_{0}$). To obtain the general expressions for each parameter, Han [36] performed a regression analysis for the experimental data of a few studies [34,37,41,42,43]. Based on the regression results, Han [36] suggested the following general equations for each parameter:

(24) $$E_{o}=42830-43T^{c}(J/\mathrm{mole})$$

(25) $$\beta =0.00017T^{c}$$

(26) $$t_{0}=0.66-0.011T^{c}\geq 0$$

(27) $$R_{u}=2.04{T^{c}}^{-0.18}\geq 1$$

where $T^{c}$ is the moist curing temperature (°C), and A is $1.0\times 10^{7}$, $2.5\times 10^{7}$, and $5.0\times 10^{7}$ for the prediction of compressive strength, splitting tensile strength, and the elastic modulus, respectively.

### 3.2. Degree of Hydration Depending on Pore Humidity

The degree of hydration $\alpha_{h}$ is closely related to the pore humidity of concrete and changes dramatically around 80% relative humidity [5,26] according to previous research based on the observations [2]. It can be modeled as shown in Equation (28), which has a similar form to the diffusion coefficient [5].

(28) $$\alpha_{h}=\frac{1}{1+{\left[(1-h)/(1-h_{c})\right]}^{n}}$$

where $\alpha_{h}$ is the degree of hydration, h is the pore humidity, $h_{c}$ is the pore humidity at $\alpha_{h}=0.5$, and n is the exponent.

**Figure 4:**
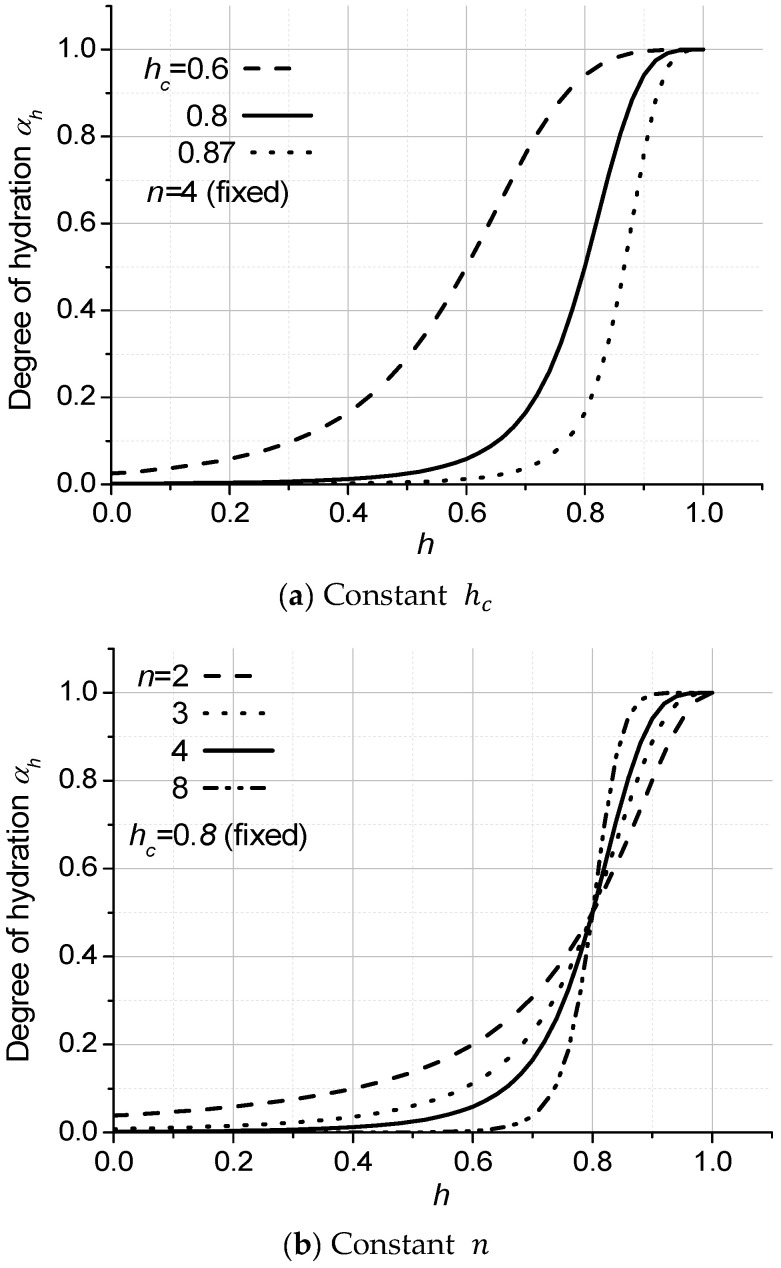
The degree of hydration depending on the parameters $h_{c}$ and n.

Figure 4 shows the variation in the degree of hydration $\alpha_{h}$ depending on the parameters $h_{c}$ and n in Equation (28). The exact relationship between the degree of hydration $\alpha_{h}$ and the pore humidity h has not been fully identified yet; therefore, appropriate values for these parameters are chosen based on the comparison of the final prediction of compressive strength with the experimental results.

### 3.3. Relationship Between Compressive Strength and Degree of Hydration

It is necessary to define the relationship between the degree of hydration and the compressive strength because it is hard to say that the development of compressive strength can be directly obtained from the degree of hydration. Therefore, the coefficient m, which represents the ratio of development of compressive strength, is introduced:

(29) $$m_{i}=\frac{∆S_{i}(h)}{∆S_{i}(h_{r})}$$

where $m_{i}$ is the ratio of the development of compressive strength at time $t_{i}$, $∆S_{i}(h)$ is the development of compressive strength at $t_{i}$ under the arbitrary pore humidity h, and $∆S_{i}(h_{r})$ is the development of compressive strength at $t_{i}$ under the reference pore humidity $h_{r}=1.0(100\%)$.

**Figure 5:**
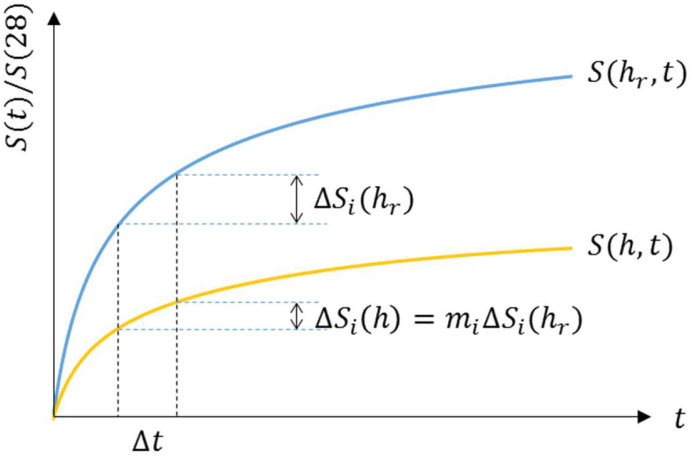
Prediction of compressive strength.

A graphical explanation of the coefficient m is represented in Figure 5. The coefficient m represents the relative development of compressive strength depending on pore humidity compared to the reference pore humidity $h_{r}=1.0(100\%)$ under moist curing.

The coefficient m becomes 1.0 when the degree of hydration $\alpha_{h}$ equals to 1.0 and decreases as the degree of hydration decreases. The relationship between the coefficient m and the degree of hydration $\alpha_{h}$ can be suggested as follows:

(30) $$m_{i}=\alpha_{h,i}^{n}$$

where $\alpha_{h,i}^{⁠}$ is the degree of hydration depending on pore humidity h at $t_{i}$, and n is the exponent.

**Figure 6:**
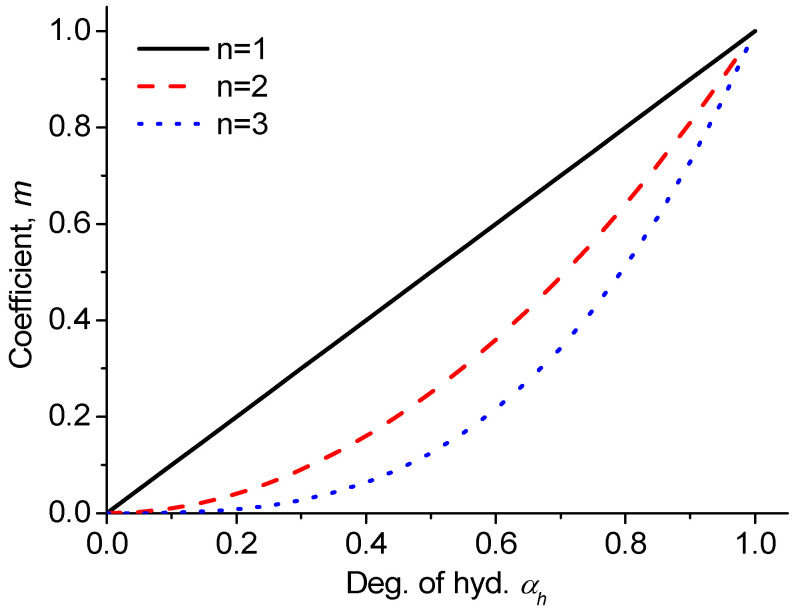
Coefficient *m* depending on degree of hydration.

Figure 6 shows the variance in coefficient m depending on the exponent n. The exact relationship between the degree of hydration and the development of compressive strength is not clearly identified; thus, the exponent n is decided based on a comparison of the analytical prediction results with the experimental results.

### 3.4. Development of Prediction Method

**Figure 7:**
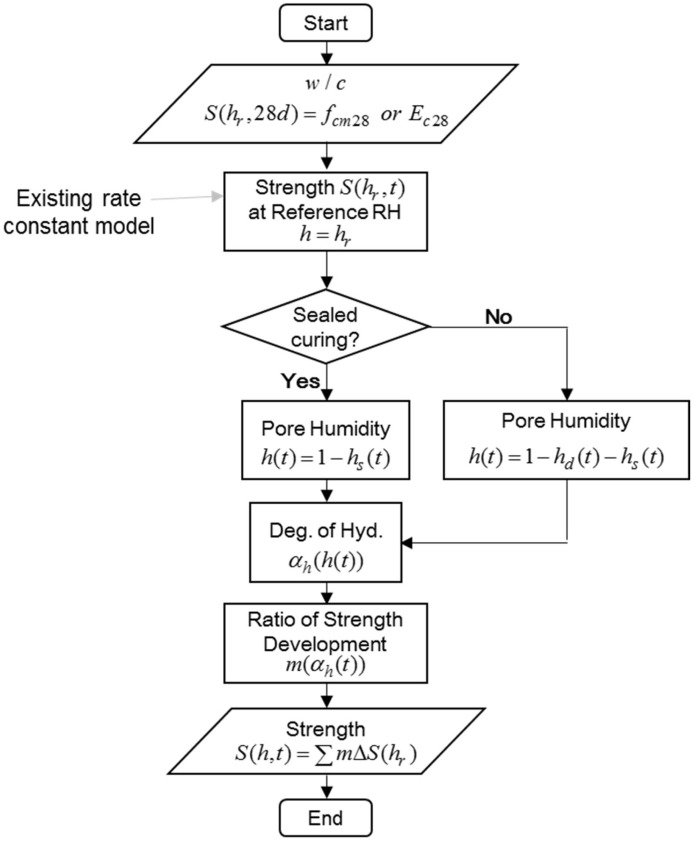
Flowchart for prediction of mechanical properties.

Figure 7 shows a flowchart for predicting the compressive strength of concrete depending on the pore humidity inside the concrete. The overall procedure follows this flowchart. Based on the input parameters, such as the water–cement ratio, compressive strength, or elastic modulus at the age of 28 days when cured under the reference humidity $h_{r}=1.0(100\%)$, the compressive strength at an arbitrary age t under $h_{r}$ can be predicted by the existing rate constant model, as shown in Equation (31), and as explained in Section 3.1.

(31) $$\frac{S(h_{r},t)}{S(h_{r},28)}=R_{u}(h_{r})\left\{1-\frac{1}{\sqrt{1+2\int_{t_{0}}^{t}k_{r}dt}}\right\}$$

where S represents the mechanical properties such as compressive strength and elastic modulus, $k_{r}$ is the rate constant at the reference humidity $h_{r}$, $R_{u}(h_{r})=S_{u}(h_{r},t)/S(h_{r},28)$, and $S_{u}$ is the ultimate (final) strength.

**Figure 8:**
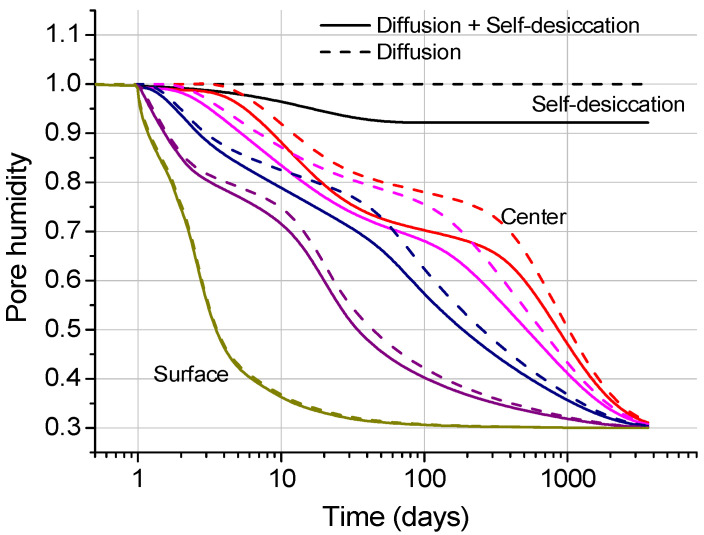
Changes in pore humidity in the cylindrical specimen due to self-desiccation and diffusion.

If the curing condition is sealed curing, then the pore humidity is the same as the humidity predicted by the new self-desiccation model shown in Equation (4). If the specimens are cured under an arbitrary humidity condition, then the pore humidity is predicted by considering both self-desiccation and diffusion. Pore humidity due to diffusion can be predicted as explained in Section 2.2. Figure 8 shows the change in pore humidity over time in the cylindrical specimen, showing the difference depending on whether the self-desiccation is considered.

Once the pore humidity h at each time step is predicted, the degree of hydration $\alpha_{h}$ can be predicted based on the relationship shown in Equation (28). The ratio of the development of compressive strength m can also be predicted based on the degree of hydration $\alpha_{h}$, as shown in Equation (30).

The final prediction of compressive strength can be obtained as follows:

(32) $$S\left(h,t\right)=\sum_{i=1}^{t}∆S_{i}\left(h\right)=\sum_{i=1}^{t}m_{i}∆S_{i}\left(h_{r}\right)$$

where $∆S_{i}\left(h_{r}\right)=S\left(h_{r},t_{i}\right)-S(h_{r},t_{i-1})$ based on Equation (31).

The purpose of the modified model is to predict the compressive strength $S\left(h,t\right)$ considering pore humidity over time when the strength of concrete at 28 days cured under the reference condition $S\left(h_{r},28\right)$ is given. This can be achieved using the developed Equations (28)–(32).

### 3.5. Verification of Prediction Method

**Figure 9:**
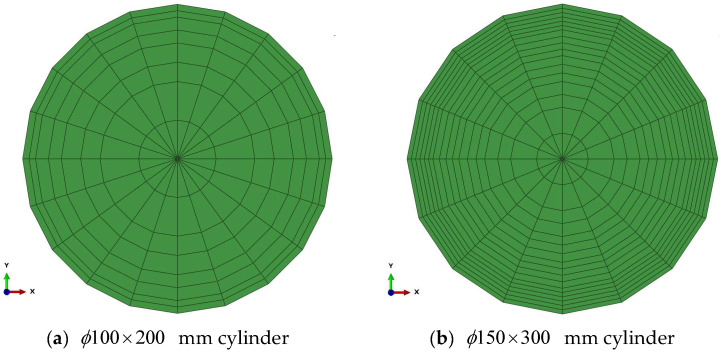
Cylinder specimens for analysis.

**Figure 10:**
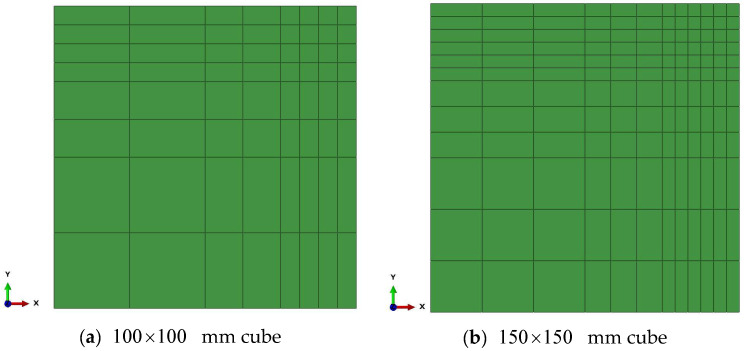
Cube specimens for analysis.

The experimental results from various studies are used to verify the modified prediction model. The shapes of the specimens are either cylinders or cubes. For the analysis, cylinder specimens are divided into several layers that become thinner at the surface, as shown in Figure 9. One-fourth of the whole cross-section is modeled for cube specimens because of the symmetry, and it is also divided into meshes, as shown in Figure 10. The pore humidity of each node over time is analyzed, and compressive strength is analyzed based on pore humidity and the modified model.

The pore humidity and compressive strength over time depend on parameters related to diffusion, such as the diffusion coefficient and surface factor. Compressive strength is also dependent on the coefficient m, $h_{c}$, and n as a function of the degree of hydration. Information on the analysis, including these parameters, is provided in Table 1 and Table 2. Table 1 shows the fixed parameters, and Table 2 shows the variable parameters depending on the mixture of concrete.

The coefficient m, $h_{c}$, and n as a function of the degree of hydration for the modified model are adopted to ensure the best fit of the analytical results with the experiments.

The parameters in Table 2 are decided from the equations explained in Section 2.2. Additionally, some experiments do not clearly denote the actual ambient humidity; hence, the condition should be assumed. Therefore, an error due to uncertainties in the diffusion coefficient or vague ambient humidity is inevitable.

**Figure 11:**
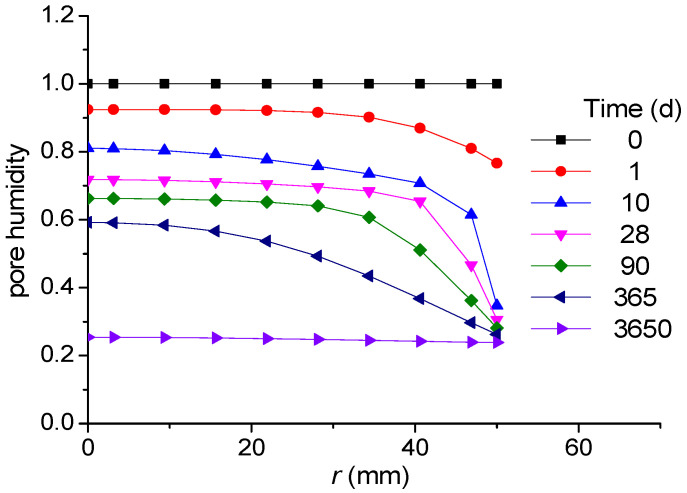
Pore relative humidity of a specimen over time (No. 4).

Ten experimental results in total are compared with the analysis results using the parameters in Table 1 and Table 2. The pore humidity values of one of the specimens resulting from the diffusion analysis is introduced in Figure 11.

**Figure 12:**
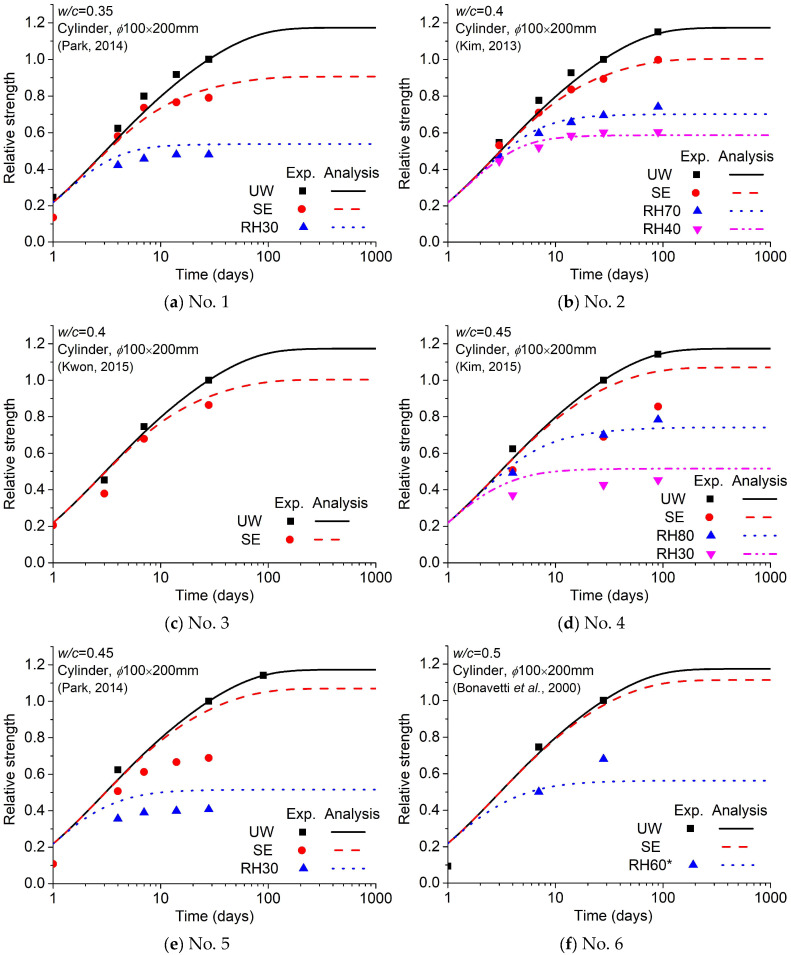

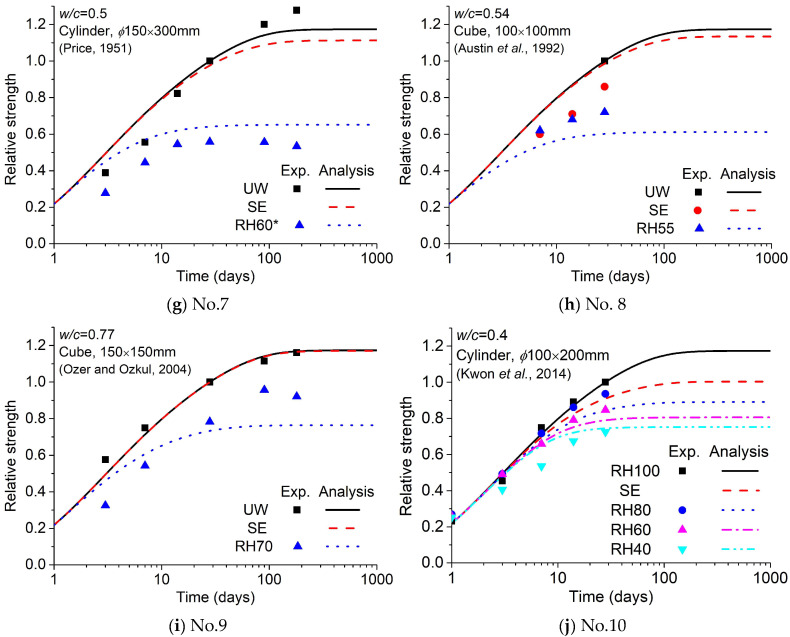
Comparison of analysis with experimental results (compressive strength) [33,44,45,46,47,48,49,50,51].

**Figure 13:**
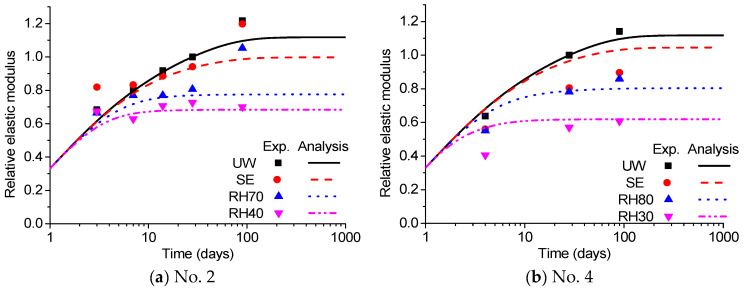
Comparison of analysis with experimental results (elastic modulus).

**Figure 14:**
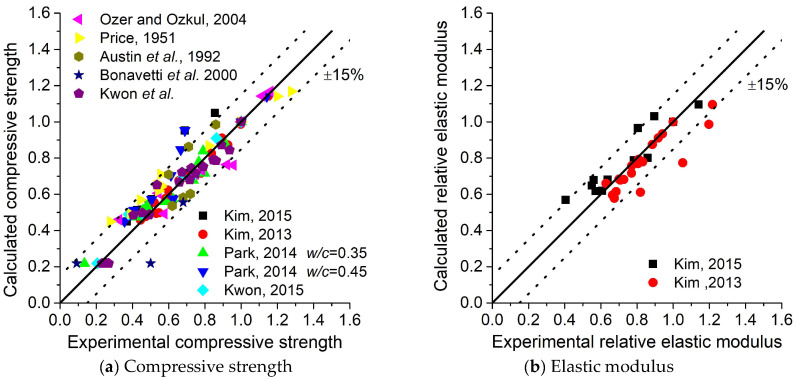
Verification of the predictive model for mechanical properties [33,44,45,46,47,48,49,50,51].

Comparisons of compressive strength are shown in Figure 12. Comparisons of the elastic modulus are shown in Figure 13. The abbreviations ‘UW’, ‘SE’, and ‘RHOO’ represent the conditions of ‘in water’, ‘sealed’, and ’OO percent of relative humidity’, respectively. A comparison of all data is shown in Figure 14, which shows that the analysis results exhibit quite good agreement with the experiments despite inevitable errors.

## 4. Conclusions

In this paper, a model to predict compressive strength is developed using experimental and analytical results based on a theoretical background. The new model is based on existing models but accounts for the effect of pore humidity on the compressive strength of concrete. The conclusions obtained in this study are as follows:

1. The degree of hydration and the development of mechanical properties change with pore relative humidity. The relationships of pore humidity with the degree of hydration and the development of mechanical properties are suggested and verified by the experimental results. The relationships show that the degree of hydration changes rapidly around 80% pore humidity, and the increase in mechanical properties at a given pore relative humidity is proportional to the cube of the degree of hydration compared to the increase under 100%% pore humidity.
2. The proposed step-by-step analysis incorporates both the concrete properties and the corresponding humidity distribution at each stage. Since the prediction model for concrete properties is based on internal humidity distribution, it can be applied to assess concrete’s properties regardless of its shape or exposure conditions, as long as the humidity distribution is available through diffusion analysis. Therefore, the proposed model is applicable to various structural sections and geometries, even those with complex or nonstandard configurations.
3. There are some assumptions for analyzing the humidity and properties of concrete in this study. In addition to comments about assumptions, some suggestions for further study are given below:
  - The self-desiccation model is an important factor that affects the strength development of sealed concrete, but it currently has some uncertainties.
  - The diffusion coefficient of concrete at an early age is one of the important factors that affect moisture diffusion and should be observed more thoroughly for various cases.

## Figures and Tables

**Table 1:** Fixed parameters for analysis.

Variable		Value
Diffusion coefficient $D\left(h,t\right)$	n	15
	$\alpha_{D}$	0.05
	$h_{c}$	0.8
Degree of hydration $\alpha_{h}$	n	2
	$h_{c}$	0.8
Coefficient, m	n	3

**Table 2:** Variable parameters for analysis.

No.	w/c	$D_{0}$ $\left(\times 10^{-7}m^{2}/h\right)$	$f_{sf}$ $\left(\times 10^{-4}m/h\right)$	Type/Size of Specimen	Ambient Humidity	Ref.
1	0.35	7.3	0.5	Cylinder/100	30%	Park (2014) [44]
2	0.4	9.7	0.5	Cylinder/100	40%, 70%	Kim (2013) [45]
3	0.4	9.7	1.0	Cylinder/100		Kwon (2015) [46]
4	0.45	9.9	1.2	Cylinder/100	30%, 80%	Kim (2015) [47]
5	0.45	9.9	1.2	Cylinder/100	30%	Park (2014) [44]
6	0.5	9.0	2.3	Cylinder/100	Air condition *	Bonavetti et al. (2000) [48]
7	0.5	8.4	2.3	Cylinder/150	Air condition *	Price (1951) [49]
8	0.54	7.1	3.2	Cube/100	55%	Austin et al. (1992) [50]
9	0.77	10	3.2	Cube/150	70%	Ozer and Ozkul (2004) [51]
10	0.4	7.7	0.1	Cylinder/100	40%, 60%, 80%	Kwon et al. (2014) [33]

* ‘Air’ condition is assumed to be 60% ambient humidity.

## Data Availability

The original contributions presented in this study are included in the article. Further inquiries can be directed to the corresponding author.
